# Utilization of a Novel Immunofluorescence Instrument Prototype for the Determination of the Herbicide Glyphosate

**DOI:** 10.3390/molecules27196514

**Published:** 2022-10-02

**Authors:** Eszter Takács, Borbála Gémes, Fanni Szendrei, Csaba Keszei, Attila Barócsi, Sándor Lenk, László Domján, Mária Mörtl, András Székács

**Affiliations:** 1 Agro-Environmental Research Centre, Institute of Environmental Sciences, Hungarian University of Agriculture and Life Sciences, Herman O. út 15, H-1022 Budapest, Hungary; 2 Institute of Isotopes Co. Ltd., Konkoly-Thege Miklós út 29-33, H-1121 Budapest, Hungary; 3 Department of Atomic Physics, Institute of Physics, Budapest University of Technology and Economics, Műegyetem rkp. 3, H-1111 Budapest, Hungary; 4 Optimal Optik Ltd., Dayka Gábor u. 6/B, H-1118 Budapest, Hungary

**Keywords:** glyphosate, competitive immunoassay, ELFIA, fluorescence detection, Project Aquafluosense

## Abstract

An enzyme-linked fluorescent immunoassay (ELFIA) method has been developed for the quantitative analytical determination of the herbicide active ingredient glyphosate in environmental matrices (surface water, soil, and plant tissues). Glyphosate, as a ubiquitous agricultural pollutant, is a xenobiotic substance with exposure in aquatic and terrestrial ecosystems due its extremely high worldwide application rate. The immunoassay developed in Project Aquafluosense is part of a fluorescence-based instrumentation setup for the in situ determination of several characteristic water quality parameters. The 96-well microplate-based competitive immunoassay method applies fluorescence signal detection in the concentration range of 0–100 ng/mL glyphosate. Application of the fluorescent signal provides a limit of detection of 0.09 ng/mL, which is 2.5-fold lower than that obtained with a visual absorbance signal. Beside the improved limit of detection, determination by fluorescence provided a wider and steeper dynamic range for glyphosate detection. No matrix effect appeared for the undiluted surface water samples, while plant tissues and soil samples required dilution rates of 1:10 and 1:100, respectively. No cross-reaction was determined with the main metabolite of glyphosate, N-aminomethylphosphonic acid, and related compounds.

## 1. Introduction

Organic micropollutants (pharmaceuticals, nutraceuticals, cosmeceuticals, pesticides, dyes, microplastics) have been identified as emerging water contaminants that present possible threats to ecological environments [1,2,3]. Agriculture is the source of various organic micropollutants, among which pesticide residues require continuous monitoring in water [4,5,6].

### 1.1. Glyphosate Herbicide Active Ingredient as an Environmental Pollutant

Glyphosate (N-(phosphonomethyl) glycine) is one of the most widely applied herbicide active ingredients worldwide. As a result of its extremely high application rates (800 thousand tons per year), glyphosate has become a ubiquitous water pollutant [7,8,9]. In spite of the somewhat favorable environmental fate parameters (DT_50_ in water and soil are 91 and 47 days, respectively), such a high load on the environment is a source of contamination by itself. It should also be noted that decomposition or dissipation can be much slower under special circumstances, e.g., in soil in the presence of numerous metal ions (Al, Fe, Mn, and Zn) due to the complexation capacity of glyphosate [10,11]. The high water solubility of glyphosate (11.6 g/L at 25 °C) is unique among pesticide active ingredients; thus, it emerges as a surface water pollutant all over the world [7,9,12], occurs in agricultural products (including maize, soybean, oilseeds, and certain vegetables, e.g., asparagus, or fruits, e.g., cherries, apricots, apples), and has been detected in biological samples, including the urine of livestock [13] and humans [14,15].

### 1.2. Analytical Methods for the Determination of Glyphosate

In recent years, several analytical methods have been developed for the quantitative measurement of glyphosate [16,17,18,19], although most of these methods require expensive and specialized instrumentation. The concentration of glyphosate in crops, produce, food and soil is usually determined by chromatographic methods [20]. These methods include a derivatization step, which depends on the analytical method and the type of detection. For gas chromatography, derivatization is necessary for reducing the polarity of the parent compound and increasing its volatility [21,22]. Derivatization is also necessary for chromatography (coupled with UV-VIS or fluorescence detection) or capillary electrophoresis methods, since the herbicide active ingredient molecule does not possess a chromophore or fluorophore suitable for measurement [17,23,24]. Even for the liquid chromatography coupled with mass spectrometry (LC-MS) method, derivatization is necessary to improve the chromatographic properties of glyphosate [25,26]. Although these methods are appropriate for glyphosate quantification in natural samples, they are costly and time-consuming procedures, especially for the measurement of a large number of samples. Glyphosate can also be determined by colorimetric methods after oxidation with hydrogen peroxide [27]. The absorbance of the phosphomolybdate heteropoly complex formed from orthophosphate is measured at a wavelength of 830 nm. Although this method provides good results, the measurement range is as high as 1–20 µg/mL, and due to the aggressive reagent hydrogen peroxide, an intense reaction may occur during the evaporation step. In a newly applied method, glyphosate is reacted with carbon disulfide, and then the dithiocarbamate group formed is used as a chelator [28]. During the chelation with copper, a yellow complex is formed, which can be easily measured at 435 nm. The disadvantage of this method is that the sample must be concentrated before measurement. In one of the latest UV-VIS detection methods [29], derivatization is carried out with 9-fluorenylmethoxycarbonyl chloride (FMOC-Cl), and absorbance is measured at 265 nm. The measurement range of the method was further reduced (84 ng/mL–21.8 µg/mL), and the measurement results were consistent with the results of the HPLC-MS/MS method used as a reference. The method is primarily suitable for the measurement of soil samples [30].

The aim of Project Aquafluosense (NVKP_16-1-2016-0049, accessed on 1 July 2022) [31] is to develop a new water analysis system for natural and artificial waters, allowing for the complex and systematic main parameter in situ assessment and monitoring of water quality by developing a modular instrument family that can be individually configured for targeted tasks at each monitoring point. The novelty of the concept is the combination of direct and immunofluorescence, along with laser spectroscopic measurement methods, with the use of optical and photonic devices. The specific research tasks of the project are: (1) the development of alternative detection methods based on fluorescence for the measurement of environmentally problematic water pollutants; (2) the expansion of the fluorescence-based detection method to measure major water quality parameters; and (3) the construction and setup of an instrument prototype for solving a complex water qualifying problem. The different modules of the instrument utilize the autofluorescence of the target water quality parameter or apply the immunoanalytical method based on fluorescence detection for water analysis. The modules are capable of measuring the (i) chlorophyll-a and -b content for the quantification of green and blue algae biomasses, respectively; (ii) aromatic amino acid tyrosine and tryptophan, which are fluorescent in the UV region and are associated with chemical and biochemical oxygen demands and total organic carbon content; (iii) polycyclic aromatic hydrocarbon (PAH) by its autofluorescence, and (iv) certain agricultural pollutants using the immunoanalytical method. The aim of the present study was to develop an enzyme-linked fluorescent immunoassay (ELFIA) module of the above instrument setup for the quantification and monitoring of the common water contaminant, herbicide active ingredient glyphosate.

## 2. Results and Discussion

Hapten-homologous competitive immunoassay protocols were devised in a heterologous phase format using colorimetric substrates with chromophores for detection, both with visual and fluorescence detection. In the immunoassay process, a protein conjugate of glyphosate immobilized on a solid surface is allowed to react with a glyphosate-specific antibody, and this immunocomplex formation process is competitively inhibited by the glyphosate content in the sample. As in the immunizing antigen glyphosate is conjugated to its carrier protein through the nitrogen atom of its glycine moiety, the resulting antiserum shows high affinity to the N-modified hapten of glyphosate; therefore, a derivatization step is applied in the immunoassays to achieve the highest utilization of the antibody affinity and avidity. The immunoassays were optimized for glyphosate determination in surface water, soil, and plant tissues.

### 2.1. Enzyme-Linked Immunosorbent Assay Development for Glyphosate

**Figure 1:**
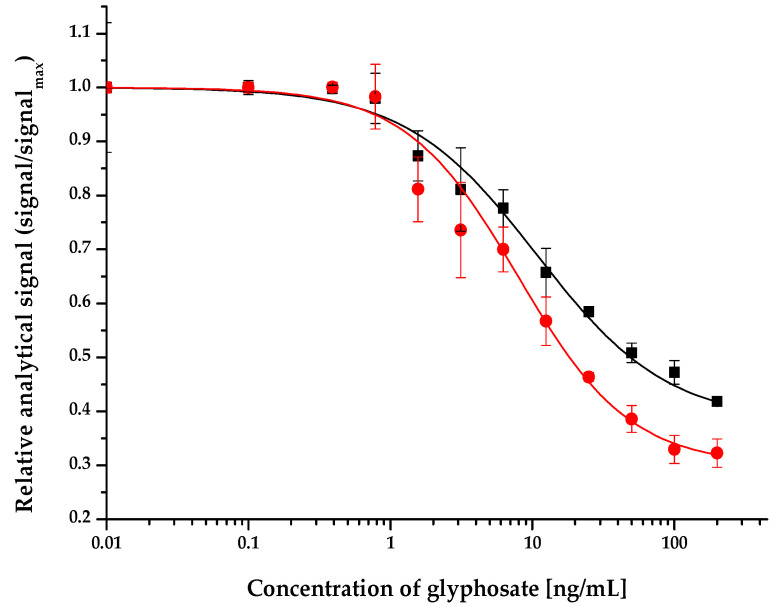
Competitive indirect calibration curves for glyphosate determined by absorbance (black) and fluorescence (red).

Indirect competitive ELFIAs were performed to established glyphosate calibration curves and to determine the limit of detection (LOD) values. LODs and statistical parameters of the calibration curves fitted by the Rodbard equation [32] in the range of 0–100 ng/mL glyphosate standard were determined for visual and fluorescence detection (Table 1). The Rodbard equation is a 4-parameter logistic regression that is a suitable evaluation method for biological systems, such as dose–response or receptor–ligand binding assays. It is generally applied in ELISA/ELFIA system characterization. The four parameters in the regression typical for competitive analytical measurements are the followings: A_1_—the upper plateau describing the background of the method where the maximum signal can be obtained; here, there is no inhibition in the binding of the glyphosate analogue immobilized onto the microplate surface and the specific antibody; A_2_—the lower plateau regarding the minimum signal at an infinite dose, where full inhibition is detected; x_0_—the point of inflection, meaning the concentration where 50% of inhibition appears (IC_50_); and p—Hill’s slope of the curve, which is related to the steepness of the curve at this point and characterizes the sensitivity of the analytical method. The LOD values were the concentration of the average analytical signal of the background (0 ng/mL glyphosate standard) minus the 3xSD (standard deviation of the background). The calibration curves and LODs were determined using both absorbance and fluorescence signals (Figure 1). For comparability of the two detection modes, assay signals are represented as relative values (signal ratios to maximal signal levels). The QuantaRed Enhanced Chemifluorescent HRP Substrate kit used for the generation of fluorescent signals contains 10-acetyl-3,7-dihydroxyphenoxazine (ADHP), a non-fluorescent compound that is dehydrogenated (oxidized) by HRP to resorufin, a highly fluorescent reaction product, which can be measured in a spectrophotometer of a microplate reader. Absorbance (optical density) and fluorescence signals were measured at 576 nm and at 593 nm wavelengths, respectively.

The ELFIA method developed in this study provides means for the determination of glyphosate with LOD values of 0.22 and 0.09 ng/mL, for visual absorbance and fluorescence, respectively, calculated based on the optical density and fluorescence signal of the negative control (0 ng/mL glyphosate standard solution). Besides the fact that detection with induced fluorescence provided 2.5-fold lower LOD values, it also provided a wider and steeper dynamic range. It should be mentioned, however, that this excellent LOD appears to be a result of the outstandingly low SD of the maximal signal (uninhibited ELFIA signal), rather than of the accuracy observed at points of partial inhibition by glyphosate.

The ELFIA developed in Project Aquafluosense is a part of an instrument family that was also constructed in a laboratory motor vehicle. Thus, the determination of glyphosate can be performed in situ. The coating and blocking steps of the microplate were carried out in the laboratory prior to immunoassay performance. In the coating process of the microplate, a glyphosate analogue conjugated to human serum albumin (HSA) was immobilized onto the polystyrene surface by passive adsorption. By setting the optimal concentration of the conjugate, desorption was negligible and did not influence the reliability of the measurement. Stabilization of the coated and blocked plates was performed using 300 µL/well of a 2% aqueous saccharose solution. Coated, blocked, stabilized, and dried microplates can be stored at 4 °C until measurement (up to approximately 1 year, if stored under dry conditions). The total immunoassay performance time, where 25 samples can be determined in parallel, is approximately 3 h (20 min derivatization, 2 h incubation with the sample and the first antibody, with washing steps, 30 min incubation with the second antibody, 2-10 min signal development). During immunoassay performance, it is necessary to cover the plates with parafilm to prevent evaporation. Adsorption of HSA can be reversed at low pH, which could potentially allow repeated use of the same microplate (upon repeated coating); however, such reuse of the plates is uncommon in ELISA/ELFIA determination protocols.

#### 2.1.1. Immunoassay Optimization

Investigation of intra-assay and inter-assay accuracy shows the general analytical consideration, namely that the reliability, accuracy, and reproducibility of the determination is not optimal towards the lower plateau of the typical sigmoid calibration curve. Thus, the standard deviation of measurement is higher near the LOD value than at the IC_50_ level, which affects the precision of the glyphosate content (Table 2). For lower concentrations, the coefficient of variability (CV) is 25.1 and 15.0 for intra- and inter-assay, respectively, while near the IC_50_ (10.75 ± 9.07 ng/mL), the CV values are 8.7 and 7.7, respectively.

#### 2.1.2. Cross-Reactivity of the Glyphosate-Specific Antibody

The specificity of the glyphosate-specific antibody (obtained from chicken) was investigated by evaluation of its reactivity to bind to various compounds structurally related to glyphosate (and also subjected to the derivatization reaction required in the immunoassay protocol). Compounds studied for cross-reactivity (CR) included the major glyphosate metabolite N-aminomethylphosphonic acid (AMPA) and other compounds with similar structures (N-(phosphonolmethyl)iminodiacetic acid or PMIDA, iminodiacetic acid, glycine, acetylglycine, sarcosine) (Table 3). The results indicate that, only at extremely high concentrations, compounds that are structurally similar to glyphosate can influence the measurements. However, these extremely high concentrations are not environmentally relevant. PMIDA at 6701 and 1650 ng/mL showed 0.013 and 0.018% inhibition of the antibody.

Determination of glyphosate in surface water, soil, and food/plant samples can be disturbed by phosphate ions; thus, inhibition of the glyphosate-specific antibody by phosphate was also required. The results indicate that the CR with phosphate is very low; however, in some cases, it is necessary to calculate using this low influence (Table 4).

### 2.2. Application of the ELFIA for Glyphosate in Environmental and Biological Samples

#### 2.2.1. Surface Water and Soil Samples

**Figure 2:**
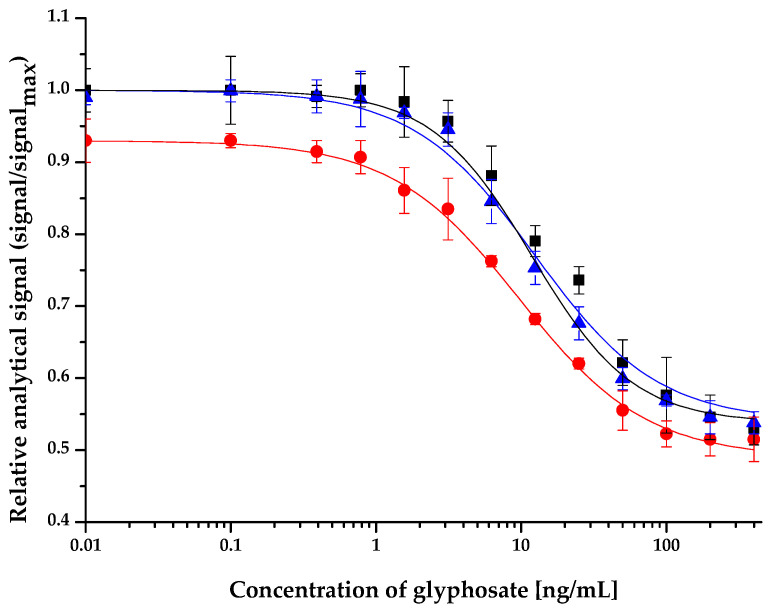
Calibration curve of the competitive enzyme-linked fluorescent immunoassay obtained in assay buffer (black), Danube surface water sample (blue), and soil diluted by 1:10 rate (red) matrices at concentration range of 0– 400 ng/mL glyphosate.

The matrix effect of 6 surface water samples (Lake Velencei at Agárd and at Pákozd, Visegrád Trout Lake and its feeding spring, Duna at Budapest, and Balaton at Tihany) and a soil sample were investigated. The calibration curves of the competitive ELFIA method at a concentration range of 0–400 ng/mL glyphosate were obtained in undiluted surface water samples and in soil samples diluted by a 1:10 ratio. The matrix effect was determined by statistical comparison of IC_50_ values of the calibration curves obtained in surface water/soil to the calibration curve obtained in the assay buffer. In the soil sample, a minor matrix effect was determined at a 1:10 dilution of the matrix compared to the assay buffer; however, this statistically significant difference disappeared at a 1:100 dilution. The IC_50_ values of the calibration curves obtained in undiluted surface water samples were not statistically different from those obtained in assay buffer (Figure 2). The IC_50_ values are presented in Table 5. As the immunoassay with fluorescence detection provides an LOD of 0.09 ng/mL and there appeared to be no matrix effect in the undiluted surface water samples, this method is appropriate for the determination of glyphosate levels below the 0.1 ng/mL authorized concentration in tap water, the official limit in the European Union of the residues of any given pesticide active ingredient in drinking water [33]. The IC_50_ values of the calibration curves in surface water and soil are summarized in Table 5.

#### 2.2.2. Biological Samples

**Figure 3:**
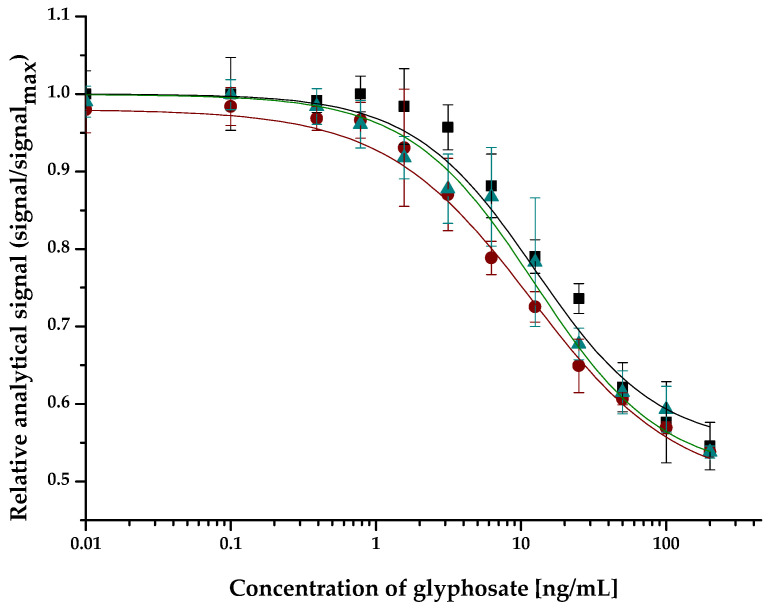
Calibration curve of the competitive enzyme-linked fluorescent immunoassay obtained in assay buffer (black), and in leaf (green) and root (brown) extracts of the plant tissue samples at a concentration range of 0 – 400 ng/mL glyphosate.

No matrix effect appeared in plant tissue samples at a matrix dilution of 1:10 ratio (Figure 3). The IC_50_ values of the calibration curve obtained in leaf and root matrices at a concentration range of 0–400 ng/mL were compared to those in the assay buffer. For the assay buffer, leaf and root extracts at 1:10 dilution IC_50_ values were 12.5 ± 0.4, 12.5 ± 0.3, and 11.8 ± 0.3, respectively. The IC_50_ values in the leaf and root extracts were not statistically different from the calibration curve in the assay buffer.

## 3. Materials and Methods

### 3.1. Materials and Reagents

Organic chemicals and solvents, glyphosate standard and structurally similar compounds, and salts for buffers were purchased from Sigma-Aldrich Inc. (St. Louis, MO, USA). The purity of standard solutions was ≥98%. The glyphosate primary antibody produced in chicken was purchased from Agrisera (Vannas, Sweden). The goat anti-chicken immunoglobulin conjugated to horseradish peroxidase (HRP) as a secondary antibody was obtained from TS Labor (Budapest, Hungary). Immunoassays were carried out in polystyrene γ-irradiated by SER-TAIN™ process sterile 96-well Costar HB microplates (Corning Inc., Corning, NY, USA) for colorimetric assay, and in low profile 96-well microplates with white wells for increased fluorescence (Bio-Rad Laboratories, Hercules, CA, USA). A QuantaRed Enhanced Chemifluorescent HRP Substrate Kit was used as the last step in the immunoassays (ThermoFisher Scientific Inc., Waltman, MA, USA).

### 3.2. Instrumentation

**Figure 4:**
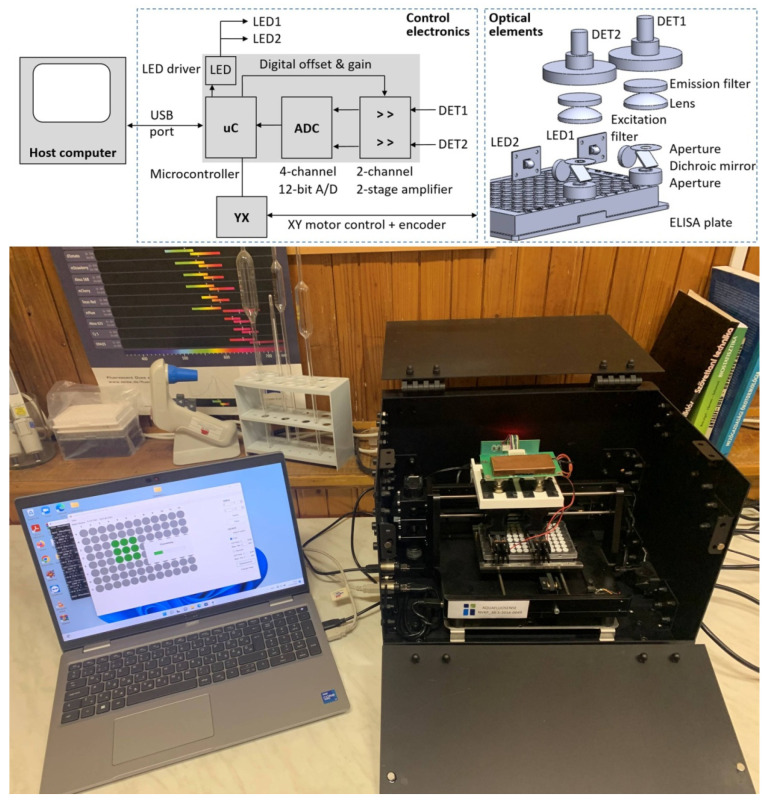
Scheme (top) and photograph (bottom) of the immunofluorescence module prototype of the induced fluorescence instrument developed in Project Aquafluosense to detect environmental pollutants and water quality parameters.

The prototype of a novel instrumentation was developed in Project Aquafluosense to detect environmental pollutants and water quality parameters using an induced fluorescent signal. One of the modular setups was elaborated to quantify organic micropollutants by immunoanalytical methods. The scheme of this immunofluorescence module is shown in Figure 4. The motor, optics and sample holder were developed at Optimal Optik Ltd. (Budapest, Hungary), while the detector electronics were created at the Budapest University of Technology and Economics (Budapest, Hungary) and described by Gémes et al. [34]. Briefly, the samples were illuminated by an LED light source with a 520 to 535 nm minimum to maximum dominant wavelength range (Cree XPEBGR-L1-0000-00F01; Cree LED, Durham, NC, USA), and the emission was detected by a dichroic beam path with silicon photodiodes (PIN-25D, OSI Optoelectronics; Hawthorne, CA, USA). Dichroic (Semrock FF562-Di03, edge: 562 nm) and bandpass optical filters (excitation: Semrock FF01-531/40-25, peak: 531 nm, width: 40 nm; emission: Semrock FF01-593/40-25, peak: 593 nm, width: 40 nm; IDEX Health & Science, Rochester, NY, USA) were applied to achieve high-spectral blocking and contrast. After the 2-stage amplification, the signal of the photodetector was fed to a 12-bit analog-to-digital converter (Analog Devices AD7864-2 with 0 to 5 V unipolar input range; Analog Devices, Wilmington, MA, USA) yielding 4095 resolvable relative fluorescence units. A traditional 96-well microplate-based immunoassay was used in a self-designed 3D-printed frame to minimize cross-talk between wells.

### 3.3. Enzyme-Linked Immunosorbent Assay

#### 3.3.1. Hapten Synthesis and Conjugation

The principle of competition in this immunoassay is the interaction between the immobilized hapten-analogue conjugate on the surface of the microplate, and the analyte in the samples. A phase heterologous competition takes place between the immobilized hapten analogue (solid phase) and the analyte in solution (liquid phase) for the binding sites of the primary antibody with the conjugate. For development of the competitive immunoassay system, the glyphosate-analogue was prepared by the Institute of Isotopes Co. Ltd. The commercially available PMIDA was reacted with succinic anhydride and conjugated to HSA. After the dialization and lyophilization of the final product, the conjugate was used for the manufacturing of the coated plate as a solid phase in this assay.

#### 3.3.2. Immunoassay

The immunoassay developed in Project Aquaflousense (EK-102M200506) was designed for the determination of glyphosate concentration in surface water and food/grain samples. Before the performance of the immunoassay, the derivatization of the standards, the control, and the samples with anhydrous acylating cocktail (acetic anhydride in acetonitrile) is required. In a polypropylene microtiter plate, 100 µL of standard/control/sample and 40 µL of the derivatization mixture are gently homogenized, then 100 µL of borate buffer is added (gently homogenization again), and the plate is incubated for 20 min. The optimal parameters and the protocol of the immunoassay are as followings: Costar HB microplates were coated with 0.5 µg/mL glyphosate-analogue conjugate. The unbound conjugate was washed 4 times with washing buffer (borate buffer). After the derivatization step, 20 µL of the derivatized standard/control/sample and 100 µL/wells of antiserum at 0.4 µg/mL concentration (chicken polyclonal anti-glyphosate as primary antibody) are incubated for *2* h at room temperature in the wells of the plate coated with glyphosate-analogue-HSA conjugate. After the incubation time, the wells are washed 4 times with 250 µL/well diluted washing buffer (borate buffer) and then 100 µL/wells of tracer (goat anti-chicken IgY–HRP as a secondary antibody) at 0.5 µg/mL concentration is added and incubated for 30 min at room temperature. Unbound secondary antibodies are washed 6 times with diluted washing buffer. For colorimetric and fluorescent detection, 100 µL/well working solution (1:50:50 *v/v* mixture of ADHP, enhancer, and peroxide) of QuantaRed Enhanced Chemifluorescent HRP Substrate Kit were added (Thermo Fisher Scientific Inc., Waltham, MA, USA). After 5 min of incubation, the enzymatic activity was stopped by 10 µL of QuantaRed Stop Solution. Absorbance of the final product resorufin was measured at its maximal absorbance wavelength, 576 nm (as recommended by the supplier [35]), by a SpectraMax iD3 Multi-Mode Microplate Reader (Molecular Devices, San Jose, CA, USA). Fluorescence was detected at 593 nm, the set wavelength for fluorescence detection that overlapped with the induced fluorescence of resorufin (as described by the supplier [35]) (see Section 3.2) by the prototype immunofluorescence module developed in Project Aquafluosense [31]. The accuracy and specificity of the immunoassay developed were also determined. Intra-assay and inter-assay accuracy were analyzed by the measurement of the samples from 1 derivatization in 10 replicates and samples from 10 independent assays in 2 replicates, respectively. Within the evaluation of specificity, the inhibition of the primary antibody by a glyphosate metabolite (AMPA), structurally similar compounds (glycine, iminodiacetic acid, PMIDA, acethylglycine, sarcosine), and phosphate as a disturbing agent were determined under optimized assay conditions.

### 3.4. Application of Immunoassay in Surface Water, Soil and Plant Tissues

Standard curves at concentration range of 0–100 ng/mL glyphosate were obtained in different surface water samples, soil, and plant tissues (leaf, root) for the evaluation of matrix effects. Surface water samples were collected from Lake Velencei at Agárd (47.190938, 18.584617) and at Pákozd (47.213213, 18.577223), Visegrád Trout Lake (47.774661, 18.986223) and its feeding spring (47.773565, 18.985176), Duna at Budapest (47.518549, 19.046216) and Balaton at Tihany (46.913958, 17.893470). The glyphosate contents of the surface water samples were determined by the chromatography method before their application in the immunoassay. Surface water samples as matrices were applied without further dilution. Soil and plant tissue samples were collected from an experiment on the environmental exposure of glyphosate in sunflower (*Helianthus annuus*) in cooperation with the Institute of Landscape Architecture, Urban Planning, and Garden Art of the Hungarian University of Agriculture and Life Sciences (Budapest, Hungary). Soil and plant tissue samples from the negative control group were collected for immunoanalytical measurement. Soil was a medium suitable for growing seedlings in trays (Klassmann Deilmann TS 3 Fine, Geeste, Germany); plant leaf and root samples were collected 5 weeks after sowing. For soil and plant tissue sample preparation, 40 mg of lyophilized ground samples were homogenized in 1 mL of phosphate buffer saline with a mortar and pestle, and centrifuged at 12,000 rpm at room temperature for 5 min. Samples were further diluted to a ratio of 1:10. The statistical analysis of standard curves was performed by the comparison of IC_50_ values using one-way analysis of variance followed by the post hoc Tukey test at a significance level of 0.05.

## 4. Conclusions

The competitive enzyme-linked fluorescent immunoassay (ELFIA) developed in this study and in the framework of Project Aquafluosense resulted in a method of improved sensitivity with a lower LOD (0.09 ng/mL) value than that in the application of the absorbance in the colorimetric assay (0.22 ng/mL). The glyphosate-specific antibody used in the immunoassay is not inhibited by AMPA (metabolite of glyphosate) and other compounds with similar structures. The LOD value of fluorescent detection provides a method for the quantification of glyphosate in surface and tap water at 0.1 ng/mL concentration, which is the maximum permitted level for a single pollutant. Thus, the immunoassay contributes to a more effective monitoring with cost-effective, in situ implementation, low LOD, and high sensitivity. Moreover, the assay is appropriate for glyphosate determination in undiluted water samples and in plant tissue and soil samples with at least 1:10 and 1:100 dilution, respectively. The 96-well microplate format allows for the measurement of 25 samples in parallel. Nonetheless, the most innovative aspect of the currently reported ELFIA lies in its application possibility with the new modular instrument setup that allows for the parallel determination of several contamination parameters of water quality.

## Figures and Tables

**Table 1:** Statistical parameters of the calibration curve obtained for a glyphosate concentration range of 0–100 ng/mL based on the Rodbard equation. Parameters are presented for absorbance and fluorescence, respectively.

Equation for Fitting: $y=\frac{A_{1}-A_{2}}{1+{\left(\frac{\text{x}}{x_{0}}\right)}^{p}}+A_{2}$ ^1^ Adjusted R^2^: 0.9859 (absorbance) 0.9895 (fluorescence)		
	Parameter	Normalized Value ± SD ^2^
Absorbance	A_1_	1.01 ± 0.01
	A_2_	0.38 ± 0.01
	x_0_	10.75 ± 1.07
	p	0.94 ± 0.11
Fluorescence	A_1_	1.01 ± 0.01
	A_2_	0.30 ± 0.02
	x_0_	7.94 ± 0.95
	p	1.10 ± 0.12

^1^ Description of the equation parameters—A_1_: upper plateau, A_2_: lower plateau, x_0_: 50% inhibition (IC_50_), p: curve slope at the inflexion. ^2^ SD: standard deviation of normalized parameter values of calibration curves.

**Table 2:** Accuracy of immunoassay developed for glyphosate determination in surface water, soil, and plant tissues.

Intra-Assay		Inter-Assay	
Average (ng/mL)	CV%	Average (ng/mL)	CV%
0.28	25.1	0.45	15.0
1.04	14.4	1.43	9.2
3.74	8	4.27	6.9
11.84	8.7	12.14	7.7

CV: coefficient of variability.

**Table 3:** Percentage of cross-reactivity (CR%) of the antiserum with glyphosate and structurally related compounds.

Compound	Nominal Concentration (ng/mL)	Detected Concentration (ng/mL)	Detected/Nominal Concentration (%)
glyphosate *	100	99.3 ± 0.8	100
	50	50.4 ± 1.1	100
AMPA *	6700	<0.1	<0.0015
	100	<0.1	<0.01
PMIDA *	6700	0.89	0.013
	1650	0.31	0.018
iminodiacetic acid	100	<0.1	<0.01
sarcosine *	100	<0.1	<0.01
glycine	6700	<0.1	<0.0015
	100	<0.1	<0.01
acetylglycine	100	<0.1	<0.01

* Chemical names: glyphosate—N-(phosphonomethyl)glycine; AMPA—N-aminomethylphosphonic acid; PMIDA—N-(phosphonolmethyl)iminodiacetic acid; sarcosine—N-methylglycine.

**Table 4:** Percentage cross-reactivity (CR%) of the antiserum with phosphate as a possible disturbing component in surface waters.

Nominal Phosphate Concentration (mM)	Detected Glyphosate Concentration (ng/mL)	Detected/Nominal Concentration (%)
175.0	17.6	<0.0015 (6.0 × 10^−5^)
87.5	8.40	<0.0015 (5.7 × 10^−5^)
43.8	4.10	<0.0015 (5.5 × 10^−5^)
21.9	2.26	<0.0015 (6.1 × 10^−5^)
	average	<0.0015 (5.8 × 10^−5^)

**Table 5:** Matrix effect in surface water and in soil extract determined by a comparison of IC_50_ values obtained in the matrices and the calibration curve in assay buffer.

**Surface Water/Soil Samples** **(GPS Coordinates)**	**Normalized IC_50_ Value (ng/mL)**	**Standard Deviation** **(ng/mL)**
assay buffer	12.5	0.4
Lake Velencei at Agárd (47.190938, 18.584617)	12.1	0.5
Lake Velencei at Pákozd (47.213213, 18.577223)	12.0	0.4
Visegrád Trout Lake (47.774661, 18.986223)	12.1	0.3
feeding spring (47.773565, 18.985176)	12.3	0.3
Duna at Budapest (47.518549, 19.046216)	11.9	0.6
Balaton at Tihany (46.913958, 17.893470)	12.0	0.4
soil sample	9.7	0.4

## Data Availability

The data presented in this study are available on request from the corresponding author. The data are not publicly available due to privacy reasons.
